# Capillaroscopic differences between primary Raynaud phenomenon and healthy controls indicate potential microangiopathic involvement in benign vasospasms

**DOI:** 10.1177/1358863X231223523

**Published:** 2024-02-09

**Authors:** Sophie Brunner-Ziegler, Eva Dassler, Markus Müller, Marco Pratscher, Nikolaus Franz-Ferdinand Maria Forstner, Renate Koppensteiner, Oliver Schlager, Bernd Jilma

**Affiliations:** 1 Department of Internal Medicine II, Division of Angiology, Medical University of Vienna, Vienna, Austria; 2 Current: Department of Clinical Pharmacology, Medical University of Vienna, Vienna, Austria

**Keywords:** microangiopathy, nailfold capillary microscopy, Raynaud phenomenon, vasospasm

## Abstract

**Background:**

For primary Raynaud phenomenon (PRP), an otherwise unexplained vasospastic disposition is assumed. To test the hypothesis of an additional involvement of distinct ultrastructural microvascular alterations, we compared the nailfold capillary pattern of patients with PRP and healthy controls.

**Methods:**

A total of 120 patients with PRP (with a median duration of vasospastic symptoms of 60 [IQR: 3–120] months) were compared against 125 controls. In both groups, nailfold capillaroscopy was performed to record the presence of dilatations, capillary edema, tortuous capillaries, ramifications, hemorrhages, and reduced capillary density and to determine a semiquantitative rating score. Further, the capacity of finger skin rewarming was investigated by performing infrared thermography in combination with cold provocation.

**Results:**

Unspecific morphologic alterations were found in both, PRP, such as controls, whereby the risk for PRP was four times as high in the presence of capillary dilations (CI: 2.3–7.6) and five times as high if capillary density was reduced (CI: 1.9–13.5). Capillary density correlated with thermoregulatory capacity in both hands in the PRP group, but not in controls. In addition, a negative correlation between the microangiopathy score and the percentage degree of rewarming in both hands was found for patients with PRP only.

**Conclusion:**

We found specific differences within the microvascular architecture between patients with PRP and controls. As a conclusion, PRP may not be an entirely benign vasospastic phenomenon, but might be associated with subtle microcirculatory vasculopathy. In addition, we suggest that the implementation of a scoring system might serve as guidance in the diagnostic process at least of patients with long-standing PRP.

## Background

Raynaud phenomenon (RP), defined by the episodic whitening of the acra upon exposure to cold, wetness, and/or emotional stress, can be classified as either primary, idiopathic, or secondary, in association with an underlying disorder.^1,2^ A pathologic entity of secondary RP has been widely recognized, especially in the area of connective tissue diseases (CTDs), yet primary Raynaud phenomenon (PRP) is generally considered a benign condition, without permanent tissue loss or general health damage.^3^ However, several reports concur that PRP may substantially compromise the quality of life of affected individuals and that its socioeconomic burden is notable, as it might interact with both private and business activities.^4^

Nailfold capillaroscopy (NC) is a noninvasive technique to reliably quantify the density and dimensions of capillaries and to describe the morphologic vascular and extravascular alterations of the digital capillary bed. The evaluation of the microvascular architecture by NC has been widely accepted as the gold standard method to differentiate between primary and secondary RP, and the implementation of specific patterns into the classification criteria of CTDs has been proposed by different studies.^5–8^

Even though reduction of blood flow might be visualized in real time by NC, objective quantification of blood flow deterioration is lacking by this method. Therefore, it is recommended that functional assessments, such as laser Doppler imaging or infrared thermography, in combination with provocation maneuvers, are added to structural NC assessment. Recently, it has been confirmed that a combination of structural and functional techniques might increase the validity of diagnostic assessment in patients with RP.^2^

Despite the underlying mechanisms of PRP not being fully clarified yet, a multifactorial pathogenesis, including an otherwise unexplained disposition for the occurrence of vasospasms, is assumed.^9^ Because investigations usually focus on the impact of environmental, humoral, and sympathetic nerve-dependent factors on the dysregulation of the vascular tone, little is known about a contribution of vascular bed abnormalities as potential pathogenetic cofactors.^10–13^

The aim of the present study was to test the hypothesis of an involvement of distinct ultrastructural microvascular alterations in the pathogenesis of PRP. For that purpose, we compared the NC pattern of patients with PRP and age- and sex-matched healthy controls in regard to capillary density, dimension, and morphology.

A secondary aim was to evaluate if the implementation of a microangiopathic scoring tool into the diagnostic assessment process of primary vasospastic disorders might be recommended as useful. We therefore analyzed if the thermoregulatory capacity in PRP was correlated with a semiquantitative microangiopathy score.^14^

## Methods

In the present study, 120 patients with PRP who were visiting the microcirculation laboratory of the Division of Angiology at the Medical University of Vienna were enrolled and compared against 125 age- and sex-matched volunteer controls. The control group consisted of individuals without any history of RP and without any clinical signs of vascular disease, who visited the clinic for routine vascular checks. At 1.5 years after the end of the inclusion period for patients with PRP, the prospectively collected study data were retrospectively analyzed. All participants provided informed written consent and the study protocol was approved by the local institutional ethics committee.

To ensure that only patients with idiopathic RP were included, we performed a comprehensive screening to exclude all those in whom any potential condition for a secondary origin for vasospasms was found. Disqualified patients included those with an occupation-related risk for vibration or chemical exposure and those whose first occurrence of vasospastic episodes was related to the intake of certain medications, drugs, or smoking. We also ruled out that participants had ever had a cold injury of their acra or suffered from disc prolapse or other vertebrogenic pain causing disorders. In addition, comprehensive laboratory assessment, including parameters of renal, thyroid, hematologic, and immunologic function and oscillometric pulse wave analysis of the extremities as well as photoplethysmographic pulse measurement of all fingers and/or toes were required to show normal results. Patients were also excluded if the capillaroscopic screening assessment revealed a scleroderma pattern or a scleroderma-like pattern, as indicated by the presence of avascular fields and giant capillaries.

**Figure 1.**
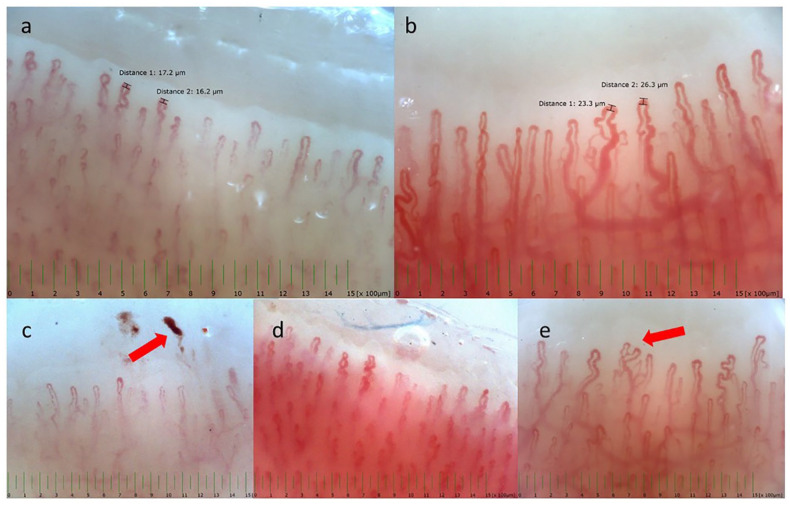
Representative pictures of nailfold capillaroscopy assessments of the study population, illustrating **(a)** normal capillary pattern, **(b)** capillary dilations, and individual morphologic features: **(c)** hemorrhages (arrow), **(d)** tortuosity, and € ramifications (off-shoots or branches; arrow).

In both study groups (PRP and control groups), general demographic and health data were recorded, such as sex, age, height, weight, body mass index, and blood pressure. In addition, NC was performed in both groups after an acclimatization period of at least 10 minutes at a constant room temperature of 21–22°C and after participants had been asked to abstain from treatment with any calcium channel blocker (CCB)-containing ointments or other local polish of the fingers and nails for 2 weeks before assessment. Using a video microscope (Optilia, Sweden), eight fingers (excluding the thumbs) were examined, capturing at least two fields of view for each finger. All finger images, magnified at 200×, were analyzed and the presence of dilatations, giant capillaries, capillary edema, tortuous capillaries (defined as the crossing of two limbs at least twice), ramifications, avascularity, and hemorrhages were assessed and recorded as dichotomous variables (present, absent). Figure 1 shows a typical normal capillaroscopic pattern and some of the most common abnormal findings.

We only recorded the most commonly reported NC parameters, without taking into account rare items, such as bushy capillaries. In a subsequent step, the capillary density was counted in a 1-mm area of all eight fingers in order to calculate the mean value. We determined a semiquantitative rating score in reference to the earlier published ‘Microangiopathy Evolution Score’, which considers the items capillary loss, capillary disorganization, and morphologic capillary alterations (0 = no changes; 1 = less than 33% changes; 2 = 33–66% changes; 3 = more the 66% changes, per linear mm) and does not include giant capillaries and hemorrhages.^14^ The microangiopathy score (MS) was finally calculated by summarizing the values of all digits and dividing by the number of examined fingers.

All NC assessments were originally performed by trained biomedical technicians. To reduce observer variability as far as possible, the stored images were then reanalyzed by a single assessor with a certified qualification in microcirculatory diagnostics, who performs approximately 30 videocapillaroscopies per month. The final findings were finally released by a single medical specialist. As the study was performed during clinical routine, it was not possible to ‘blind’ the technicians concerning Raynaud patients or healthy controls as technicians are usually informed about the assignment diagnosis of those patients visiting the outpatient clinic. Also, patients often are happy to talk about their complaints during investigations.

In a subgroup of participants, an additional infrared thermographic assessment of the finger skin temperature in combination with a cold provocation test was performed. Detailed steps of this assessment procedure have been described previously.^3^ In brief, after an acclimatization period of 10 minutes at constant room temperature, the patients were instructed to place their hands with the palm side up at a fixed distance from an infrared thermography camera (Thermo Tracer TH1 100; San-EI), which was placed on a tripod. A total of three images of each hand were captured and all temperature maps were achieved using an attached computer. Acral skin temperature was determined from the center of each fingertip using the device-related software program (PicWin-IRIS, version 6.5) and average values were reported for the right and the left hands. With the first images, the basal finger skin temperature of both hands was determined. If the fingers were warmer than 30°C, an external cold provocation was performed by placement of the hands into a water-bath for 1 minute (20°C for the PRP group and 6°C for the controls). The patients always wore thin hand gloves to prevent evaporation. Immediately after the cold provocation, second images were taken followed by a rewarming period of 10 minutes until the third and last images for final skin temperature measurements were obtained. The capacity of rewarming, expressed as the percentage difference between rewarming and cooling temperatures, was used as the main outcome variable. This parameter was calculated by subtracting cooling (C) values from rewarming (R) values, multiplying by 100 and dividing by the difference between basal (B) and cooling values ( $\frac{:R-C)*100}{\left(B-C\right)}$ .

### Statistical analysis

Descriptive data are reported as mean with SD and median with IQR for continuous variables and frequency and percentage for categorical data. Counts were presented as numbers and proportions. For the comparison between PRP and controls, mean differences were tested by *t*-test for independent samples. Categorical variables were compared by applying the Pearson’s chi-squared test. Correlations between capillary characteristics and the two study groups were primarily explored by univariate analysis and significant results were subsequently tested with a multivariate logistic regression model, adjusting for sex and using the control group as the categorical reference. Correlations of metric variables were assessed groupwise using Pearson’s correlation. Unless otherwise indicated, *p* ⩽ 0.05 was considered statistically significant. IBM SPSS Statistics, version 29 (Armonk, NY, USA) was used for all analysis.

## Results

A total of 245 participants were included (120 with PRP and 125 controls) for data and NC assessment. Out of these, 173 (50 with PRP and all [*n* = 125] controls) agreed to additionally take part in an infrared thermographic assessment of the finger skin temperature in combination with a cold provocation test. Table 1 summarizes the demographic data and clinical features of the study population, including thermographically assessed basal skin temperature and degree of rewarming after cold provocation of both hands. Though there was no difference regarding age and sex between individuals with PRP and controls, patients with PRP had significantly lower systolic and diastolic arterial blood pressure levels, as well as significantly lower body mass index, than controls (matching the characteristic ‘PRP phenotype’). At basis, the mean values of the average finger skin temperature were significantly lower in patients with PRP than in controls in both hands.

**Table 1.** Demographics and clinical characteristics of the study population separated into the two study groups.

	Healthy volunteers (*n* = 125)	Patients with PRP (*n* = 120)	*p*-value^a^
Age, years, mean (SD)	47.9 (14.4)	45.5 (15.8)	0.22
Women, *n* (%)	90 (72)	85 (71)	0.84
Duration since first occurrence of Raynaud symptoms, months, median (IQR)	–	60 (3–120)	
Systolic arterial blood pressure, mmHg, mean (SD)	132 (17)	129 (17)	0.001
Diastolic arterial blood pressure, mmHg, mean (SD)	83 (11)	75 (10)	0.001
Body mass index, mean (SD)	26.1 (5.5)	22.1 (3.5)	0.001
Average basal skin temperature of fingers of right hand, degrees, mean (SD)	30.7 (3.6)	27.5 (4.8)	0.001
Average basal skin temperature of fingers of left hand, degrees, mean (SD)	30.6 (3.6)	27.2 (4.7)	0.001
Degree of rewarming of right hand, %, mean (SD)	83.3 (36.2)	64.6 (129.1)	0.16
Degree of rewarming of left hand, %, mean (SD)	83.2 (35.3)	81.2 (154.8)	0.90

a Reported *p*-values refer to the assessment of heterogeneity between healthy volunteers and patients with PRP.

PRP, primary Raynaud phenomenon.

In PRP, the median duration since first occurrence of Raynaud symptoms (calculated as the time interval between symptom onset and the date of NC assessment) was 60 months (IQR: 3–120 months). In general, the number of prescription drugs was low in both study groups. Overall, the most often reported medication was lipid-lowering drugs and only a very limited number of participants with PRP reported the intake of antihypertensive drugs, but always for reasons of cardiovascular risk control and not for the purpose of counteracting acral vasospasms. Comparison of NC assessments between PRP and controls, including frequencies of distinct parameters and the semiquantitatively calculated MS, is displayed in Table 2. Unspecific morphologic alterations could be found in both, in PRP, such as in controls (Figure 1); however, the presence of a scleroderma pattern was excluded in all participants by the absence of avascular fields and giant capillaries.

**Table 2.** Univariate and odds ratio analyses of capillaroscopic findings for the two study groups.

	Healthy volunteers *n* = 125 (%)	Patients with PRP *n* = 120 (%)	*p*-value^a^	Odds ratio^b^, 95% CI	
Presence of dilated capillaries	57 (45.6)	94 (78.3)	0.001	4.15	2.27–7.56
Reduced capillary density (< 7/linear mm)	6 (4.8)	25 (20.8)	0.001	5.00	1.86–13.46
Capillary density (µm)/linear mm, mean (SD)	7.95 (0.8)	7.65 (9.9)	0.009		
Presence of edema	0	6 (7.3)	0.002		
Presence of tortuous capillaries	61 (48.8)	57 (47.5)	0.79		
Presence of ramifications	28 (22.4)	20 (16.7)	0.25		
Presence of microhemorrhages	31 (24.8)	45 (37.5)	0.03	1.32	0.71–2.48
Microangiopathy score, mean (SD)	0.30 (0.3)	0.36 (0.3)	0.03 (one-sided)		

a Reported *p*-values refer to the assessment of univariate heterogeneity between healthy volunteers and patients with PRP.

b Odds ratios of multivariate logistic regression analyses of significant univariate results, adjusted for sex and using the control group as categorical reference.

PRP, primary Raynaud phenomenon.

In univariate analysis, the proportions of ramifications and tortuous capillaries did not differ between groups. Conversely, the parameters edema, microhemorrhages, and dilated capillaries were significantly more frequent in patients with PRP than in controls (Figure 1). In addition, the mean capillary density/mm was significantly lower in patients with PRP than in controls. Similarly, when capillary density was expressed as a categorical variable according to a normal value of seven capillaries/mm, the reduction of capillary density was more frequent in patients with PRP than in controls. In patients with PRP, mean capillary density was correlated to percentage degree of rewarming after cold provocation in both hands, whereas there was no relationship between density and thermoregulatory capacity in healthy controls. Capillary density was correlated to age in both groups.

In the multivariate logistic regression analysis, the following parameters remained statistically significantly associated with PRP, even after controlling for sex: dilated capillary and reduced density. Specifically, the odds of suffering from PRP were four times as high in the presence of capillary dilations (CI: 2.3–7.6) and five times as high if capillary density was reduced (CI: 1.9–13.5; Table 2).

With respect to the MS, a tendency for higher values was recorded in patients with PRP than in controls; however, with borderline significance (on a one-sided level) only.

An overview of significant correlations between demographic, structural, and functional assessment parameters in patients with PRP is illustrated in the online supplemental material. In both hands, initial temperature values, such as rewarming capacity, correlated between the right and left sides. As expected, the MS correlated negatively to capillary density. Capillary density additionally correlated to age and the degree of rewarming of both hands in the PRP group, whereas there was no relationship between density and thermoregulatory capacity in heathy controls. Age further correlated with the basal finger temperature of both hands in patients with PRP and also to a weaker extent (not shown) in controls. A statistically significant but weak association between the MS and the percentage degree of rewarming in both hands was found in the PRP group, but not in controls.

## Discussion

In the present investigation, we found specific differences within the microvascular architecture between PRP and controls. Within the study cohort, distinct features, such as presence of dilated capillaries and/or reduced capillary density, increased the likelihood for PRP.

For secondary RP, several reports have published NC classification criteria to distinguish between underlying disorders, such as scleroderma disease,^15–18^ and microangiopathy scores have been proposed not only to quantify disease severity, but also to monitor a potential progression.^14^ However, attempts to identify a distinct capillaroscopic pattern, specifically describing a Raynaud disorder of primary origin, are rare and there is no information on whether the potential implementation of any scoring system into the diagnostic assessment of ‘benign vasospasms’ might be recommended.

Early hypotheses on the involvement of structural microvascular changes in PRP were based on the finding of increased capillary dimensions in individuals with PRP in comparison to controls.^19^ However, this study included only nine patients with PRP and an older capillaroscope was used instead of an up-to-date applied modern extended videocapillaroscopic method. Since then, further investigations, primarily focusing on secondary RP and using patients with PRP as reference, have confirmed the finding of diverse microvascular alterations in the primary form. However, data on their incidence and nature are rare, but could be important to better understand some of the multifactorial pathogenetic mechanisms of this disorder.^20^ In the same way, incidental data on NC assessment in healthy individuals, disclosing potential capillary alterations, are scarce as well.^21^

Distinguishing patients with PRP from healthy individuals and verification of diagnosis is not only important for the condition to be accepted (acknowledged), but also in terms of recommendations that can be given to the patient.

Interestingly, nonspecific dilations are not included, either in the PRINCE (prognostic index for nailfold capillaroscopic examination) index, or in the Microangiopathy Evolution Score, both of which are used as tools to predict or monitor a scleroderma spectrum disorder.^14,22^ Nevertheless, in a more recent publication on secondary RP, high numbers of dilated capillaries at basal investigation were attributed to an increased risk for evolving vascular damage.^14^ In our report, we interpret increased numbers of capillary dilations in PRP as an indication for elevated intraluminal pressure, resulting from a disbalance between arteriolar vasoconstriction and dilation.^23,24^ In the PRP group, the median duration since the first occurrence of vasospastic symptoms was quite long. However, as the present study does not include any follow-up investigations, we are not able to differentiate if reduced capillary density in our patients with PRP has been directly contributing to the impaired capillary function (and resulting clinical symptoms) or if the capillary loss rather reflects the secondary result of an impaired adaptive capacity of dysfunctional capillaries. Because all our PRP patients displayed normal perfusion results in both the upper and the lower leg extremities, a reduction of capillary numbers does not seem to be attributed to peripheral artery disease.

The prevalence of microhemorrhages was higher in patients with PRP than in controls, at least in univariate analysis. In earlier publications, it was argued that a high frequency of hemorrhages in PRP reflects a predisposition of the endothelium for hemorrhagic rather than thrombotic events.^25^ In corroboration, we could rule out vascular damage on the basis of atherothrombosis, as already discussed above. In contrast to earlier suggestions,^21^ we do not believe that nailfold hemorrhages are the result of external manual damage because individuals with suspected vibration-induced white finger syndromes were excluded from the present analysis.

From our preliminary findings, it does not seem legitimate to draw final conclusions and claim solitary findings to be the main and only pathogenetic mechanisms. Other parameters coming out less strong might also play a role. In accordance with others, we rate ramifications and tortuous capillaries, which could be found in comparable frequency in both study groups, as unspecific findings without pathognomonic relevance;^21^ however, it might be hypothesized that the association with vasospastic tendencies could become stronger by the entire spectrum of microvascular peculiarities.

In regard to the paucity of NC studies, visualizing morphological characteristics and calculating capillary density in healthy individuals, one large cross-sectional study on 150 healthy individuals reported the presence of tortuous capillaries in 45%, ramified capillaries in 7%, and reduced density in 33%.^26^ On the other hand, Hoerth et al. found tortuous capillaries in 43%, ramified capillaries in 47%, and age-dependent reduced density in around 18–40% in a total of 120 volunteers.^21^ Even if proportions of distinct alterations differ between these earlier studies and also in comparison to our results, we confirmed a high percentage of deviating capillaries in a healthy population. Therefore, it seems even more interesting to discover major differences of distinct microvascular features between individuals with and without PRP. With respect to publications on capillary density, the comparison of study results is limited by authors using different cut-off values for the categorization of a ‘regular capillary density’. In accordance with a recent manuscript, we found a positive correlation between capillary density and age in both study groups.^26^ Besides, various sex-dependent morphological and functional abnormalities of nailfold capillaries have been reported not only of patients with collagenosis but also in healthy individuals. In this context, it has been shown that ectasia and reduced capillary filling is more common in women, whereas tortuous capillaries are more prevalent in men.^27^ As far more women than men have been included in the present analysis, we were not able to analyze sex-specific peculiarities.

It was suggested that for patients suspected of capillary dysfunction, the inclusion of an at least semiquantitative scoring of NC assessment results should be recommended.^14^ On the other hand, it was argued that application of a scoring system, developed for scleroderma patients, is of questionable value for healthy subjects.^21^ In the present investigation, we found higher MS values for participants with PRP than for those without vasospasms. Even if the difference reached borderline significance only, application of a quantification tool might facilitate objective differentiation. Suitability of the use and value of the MS in PRP must be further assessed in prospective studies on larger patient numbers.

Recently, it has been proposed that a combination of structural and functional methods might increase the validity of diagnostic assessment in patients with RP;^2^ however, little information is known on potential relations between microvascular structures and thermoregulatory function in patients with RP without CTD manifestations. Therefore, we performed correlation analyses of the assessment results of NC and infrared thermography in combination with cold provocation in patients with PRP in comparison to controls.

As expected, patients with PRP had significantly lower finger skin temperatures at baseline than controls in both hands. Basal temperature values correlated with age in both groups, which is in contrast to a former publication on a smaller study population, which described such a correlation only for those with PRP and not for controls.^3^ However, in the interpretation of thermoregulatory performance studies (with and without the combination of cold provocations), it has to be considered that the diagnostic validity is highly dependent on the possibility of controlling thermal conditions during the examination.^28,29^ In our patients with PRP, the thermoregulatory capacity of both hands, expressed as percentage rewarming after cooling, was positively correlated to mean capillary density and (albeit weakly) negatively correlated to MS, whereas there were no such relations in healthy controls. These findings serve as a further indication that structural mechanisms might be involved in conditions of impaired thermoregulation, at least on the basis of capillary vasospasms.

### Limitations of the study

First, it is a monocentric study. Second, we did not calculate the average dimensions of visible capillaries of individuals, but only assessed the presence or absence of dilated capillaries as a categorical variable. Further, we cannot rule out that Raynaud symptoms of primary origin might evolve into secondary forms later on. For that reason, the study results were retrospectively analyzed 1.5 years after the end of the inclusion period for primary Raynaud patients, who all were instructed to schedule a follow-up visit in case of deterioration of symptoms and/or the occurrence of any symptoms compatible with an immunologic disease. We are further aware that the median duration since first occurrence of Raynaud symptoms was quite long, and based on earlier studies and also on our own experience from our microcirculation laboratory we rate the risk for conversion into a secondary form highest within the very first year of the beginning of symptoms. Patients who reported an initial onset of symptoms within less than 1 year were instructed to repeat their immunologic assessment within the following months and to return in case of conspicuous results. Additionally, patients were only included if their immunologic blood test at the time of NC assessment was negative; in particular, if antinuclear antibody titers were not elevated to more than 1:160 and their NC exploration was free of giant capillaries and vascular fields. None of the patients was treated with methotrexate, cyclosporin, or other medications, which have incidentally been reported to induce NC changes.^14,30^ Last, as the median duration since first occurrence of Raynaud symptoms was not correlated to any of the structural or functional assessment parameters, the definition of long-lasting, stable Raynaud disorder of idiopathic origin seems to be justified for our cohort.

## Conclusion

In conclusion, PRP seems to be associated with a peculiar microvascular pattern, including increased capillary dimensions and capillary loss. The illustration of NC differences between healthy subjects and patients with PRP improves the diagnostic specificity of microcirculatory assessment and might further contribute to identifying potential mechanisms of a microvascular involvement in primary Raynaud. Based on the present results, we hypothesize that PRP may not be an entirely benign vasospastic phenomenon but might be associated with subtle microcirculatory vasculopathy. In addition, we confirmed that that thermoregulatory capacity of PRP significantly differs in comparison to individuals without vasospasms and suggest that the implementation of a scoring system might serve as guidance in the diagnostic process, at least of patients with long-standing PRP. Prospective long-term studies are warranted to validate the use and applicability of this score in daily routine practice.

## Supplemental Material

sj-pdf-1-vmj-10.1177_1358863X231223523 – Supplemental material for Capillaroscopic differences between primary Raynaud phenomenon and healthy controls indicate potential microangiopathic involvement in benign vasospasms Supplemental material, sj-pdf-1-vmj-10.1177_1358863X231223523 for Capillaroscopic differences between primary Raynaud phenomenon and healthy controls indicate potential microangiopathic involvement in benign vasospasms by Sophie Brunner-Ziegler, Eva Dassler, Markus Müller, Marco Pratscher, Nikolaus Franz-Ferdinand Maria Forstner, Renate Koppensteiner, Oliver Schlager and Bernd Jilma in Vascular Medicine
