# Supplementary material for: Capillaroscopic differences between primary Raynaud phenomenon and healthy controls indicate potential microangiopathic involvement in benign vasospasms
Source: Vasc Med. 2024 Feb 9;29(2):200–7. doi: 10.1177/1358863X231223523 (PMC11010550; doi:10.1177/1358863X231223523)
Supplement: sj-pdf-1-vmj-10.1177_1358863X231223523 – Supplemental material for Capillaroscopic differences between primary Raynaud phenomenon and healthy controls indicate potential microangiopathic involvement in benign vasospasms [file sj-pdf-1-vmj-10.1177_1358863X231223523.pdf]

Supplemental Material

Table 1 Suppl Overview on Pearson´s correlations between demographic, structural and functional assessment parameters in patients with PRP

**Pearson Correlation Coefficient; p-value**	**Age, mean years (SD)**	**Capillary density (µm)/linear mm, mean (SD)**	**Microangio-pathy Score, mean (SD)**	**Average basal skin temperature of the right hand fingers, mean degree (SD)**	**Average basal skin temperature of the left hand fingers, mean degree (SD)**	**Degree of rewarming of the right hand, percentage (SD)**	**Degree of rewarming of the left hand, percentage (SD)**
**Age, mean years (SD) Age, mean years (SD)**		*0.28 P=0.002*		*0.46 P=0.001*	*0.46 P=0.001*		
**Capillary density (µm)/linear mm, mean (SD)**	0.28 P=0.002		*-0.52 P=0.001*			*0.49 P=0.005*	*0.54 P=0.002*
**Microangiopathy Score, mean (SD)**		-0.52 P=0.001				*-0.39 P=0.03*	*-0.39 P=0.03*
**Average basal skin temperature of the right hand fingers, mean degree (SD)**	0.46 P=0.001				*0.97 P=0.001*		
**Average basal skin temperature of the left hand fingers, mean degree (SD)**	0.46 P=0.001			0.97 P=0.001			
**Degree of rewarming of the right hand, percentage (SD)**		0.49 P=0.005	-0.39 P=0.03				*0.96 P=0.001*
**Degree of rewarming of the left hand, percentage (SD)**		0.54 P=0.002	-0.39 P=0.03			0.96 P=0.001	

Only correlation coefficients whose corresponding p-values reached significance on a two-sided basis are illustrated
SD: Standard Deviation
